# Identification of Adipsin as a Biomarker of Beta Cell Function in Patients with Type 2 Diabetes

**DOI:** 10.3390/jcm13237351

**Published:** 2024-12-02

**Authors:** Jae-Hyung Park, Thi Nhi Nguyen, Hye Min Shim, Gyeong Im Yu, Eun Yeong Ha, Hochan Cho

**Affiliations:** 1 Department of Physiology, Keimyung University School of Medicine, Daegu 42601, Republic of Korea; physiopark@kmu.ac.kr (J.-H.P.); 1114177@stu.kmu.ac.kr (T.N.N.); shm85@dsmc.or.kr (H.M.S.); rki0411@hanmail.net (G.I.Y.); 2 Department of Internal Medicine, Keimyung University School of Medicine, Daegu 42601, Republic of Korea

**Keywords:** adipsin, biomarker, beta cells, type 2 diabetes

## Abstract

**Background/Objectives**: Adipsin, an adipokine, is known to play an important role in maintaining the function of pancreatic beta cells in mice. This study aimed to investigate whether adipsin could be a circulating biomarker for evaluating the function of beta cells in patients with type 2 diabetes (T2D). **Methods**: Plasma adipsin concentrations were measured using immunoassay in three distinct subject groups: normoglycemia, T2D without insulin treatment (T2D-w/o-insulin), and T2D treated with insulin (T2D-with-insulin). Adipsin expressions were evaluated in three distinct mouse groups: normal diet (ND), high-fat diet (HFD), and HFD with streptozotocin (STZ) and nicotinamide (NA). **Results**: The T2D-with-insulin group exhibited a significant decrease in plasma adipsin concentration (3.91 ± 1.51 μg/mL) compared to the T2D-w/o-insulin group (5.11 ± 1.53 μg/mL; *p* < 0.001), whereas the T2D-w/o-insulin group showed a significantly increased plasma adipsin concentration compared to the normoglycemia group (4.53 ± 1.15 μg/mL). Plasma adipsin concentration was positively correlated with fasting C-peptide level (*p* < 0.001), 2-h C-peptide level (*p* < 0.001), and 2-h C-peptidogenic index (*p* < 0.001) in the diabetic groups. HFD mice showed a significant increase in pancreatic islet size, plasma insulin and adipsin levels, as well as adipsin expression in white adipose tissue (WAT) compared to ND mice. In contrast, the insulin-deficient T2D model (HFD-STZ-NA) demonstrated a marked reduction in pancreatic islet size, plasma insulin and adipsin concentrations, and adipsin expression in WAT compared to the HFD mice. **Conclusions**: plasma adipsin may be useful for evaluating pancreatic beta cell function in patients with T2D.

## 1. Introduction

Diabetes mellitus is a group of metabolic disorders characterized by chronic hyper-glycemia resulting from defects in insulin secretion, insulin action, or both. This condition leads to disturbances in carbohydrate, fat, and protein metabolism. The diagnostic criteria for diabetes are as follows: (1) fasting plasma glucose (FPG): a level of ≥126 mg/dL (7.0 mmol/L) after no caloric intake for at least 8 h; (2) 2-hour plasma glucose (2-h PG): a level of ≥200 mg/dL (11.1 mmol/L) during an oral glucose tolerance test (OGTT), which involves consuming a glucose load containing the equivalent of 75 g anhydrous glucose dissolved in water; (3) glycated hemoglobin (A1C): a level of ≥6.5% (48 mmol/mol); and (4) random plasma glucose: in individuals with classic symptoms of hyperglycemia or hyperglycemic crisis, a random plasma glucose level of ≥200 mg/dL (11.1 mmol/L). In the absence of unequivocal hyperglycemia, a diagnosis requires two abnormal test results from the same sample or in two separate test samples [1].

According to data released in 2021, 537 million adults (from 20 to 79 years old) have diabetes, which is about 1 in 10. Diabetes is expected to rise to 643 million by 2030 and to 783 million by 2045. More than three in four adults with diabetes live in low- and middle-income countries. Diabetes caused 6.7 million deaths, one every 5 s, in 2021. Diabetes has cost at least $966 billion in healthcare spending, a 316% increase over the past 15 years. In addition, 541 million adults suffer from impaired glucose tolerance (IGT), which puts them at high risk for T2D [2]. Health complications related to diabetes pose a significant financial burden on individuals and society. The annual cost of being diagnosed with diabetes in the United States in 2022 was estimated to be $413 billion, including $307 billion in direct medical costs and $106 billion in reduced productivity. After adjusting for inflation, the economic cost of diabetes increased by 7% between 2017 and 2022 and 35% between 2012 and 2022 [3,4]. In the Republic of Korea, data from 2020 revealed that 16.7% of adults aged 30 and above have diabetes. The prevalence is higher among men (17.7%) compared to women (13.6%). Notably, among individuals aged 65 and older, the prevalence increases to 30.1% [5]. These statistics underscore the growing global and national burden of diabetes, highlighting the need for effective prevention and management strategies.

Biomarkers play a crucial role in assessing pancreatic beta cell function in individuals with T2D, helping to monitor disease progression and therapeutic efficacy [6]. Traditional biomarkers such as fasting glucose, insulin, and C-peptide levels have long been used to evaluate beta cell activity and insulin resistance [7]. Recent studies are expanding the scope of biomarkers, including genetic markers, circulating molecules like proinsulin, and protein biomarkers, that may better predict beta cell dysfunction and progression toward diabetic complications [8,9]. Developing novel biomarkers to assess beta cell function in T2D could enable earlier detection of beta cell decline, improve treatment personalization, and better predict disease progression.

Adipsin, an adipokine secreted from adipose tissue, plays an important role in the complement pathway [10]. It is an integral player in the alternative complement pathway, where it cleaves c3 into c3a and c3b, contributing to immune function [11,12]. C3a, a downstream product of this enzymatic reaction, has been implicated in both anti-inflammatory and pro-inflammatory effects and has been reported to upregulate the function of antigen-presenting cells, including dendritic cells [13]. These findings suggest that adipose tissues, known for their high expression of adipsin [14], play a crucial role in the immune system.

Adipose tissue also plays an important role in metabolic regulation and disease progression [15,16]. However, the metabolic regulation of adipose tissue through adipsin is not well known. Recently, it has been revealed that adipose tissue plays an important role in the preservation of beta cells and in the regulation of beta cell function through adipsin. Notably, Lo et al. [17] demonstrated that adipsin-null mice displayed a defect in insulin secretion as well as glucose-induced insulin secretory response; and that adipsin stimulates insulin secretion via the C3a receptor pathway in beta cells. C3a is a complement factor produced by adipsin in the blood, and C3a increased insulin secretion by increasing intracellular ATP and Ca^2+^ levels in beta cells. Additionally, they found that adipsin levels in the plasma and adipose tissue of diabetic mice were lower than in normal mice, and restoration of adipsin ameliorated hyperglycemia by enhancing insulin secretion in diabetic mice. Gómez-Banoy et al. [18] demonstrated that chronic restoration of adipsin using gene transfer substantially preserved beta cell mass in diabetic mice by reducing dual specificity protein phosphatase 26 (Dusp26). Depletion of Dusp26 by adipsin/C3a increased expression of key beta cell identity genes and prevented apoptosis. These findings suggest that adipsin plays an important role in beta cell survival and function and may be a potential therapeutic target for T2D.

However, it is still unclear whether plasma adipsin can be a biomarker reflecting beta cell function in patients with T2D. Therefore, we investigated the association between plasma adipsin and clinical parameters to determine the usefulness of measuring plasma adipsin concentration as a biomarker for evaluating beta cell function in patients with T2D. Furthermore, we utilized insulin-deficient T2D mice to explore the relationship between adipsin expression in white adipose tissue (WAT) and pancreatic beta cell mass.

## 2. Materials and Methods

### 2.1. Study Design and Clinical Parameters

The study participants for the evaluation of plasma adipsin levels were recruited between March 2020 and March 2022 at Keimyung University Dongsan Hospital. The criteria for selecting subjects were adults aged 19 or older, and criteria for each group is listed below. The criteria for selecting the normoglycemia group are those who have three or more Korean nationalities and have lived in Korea, and the criteria for exclusion are as follows: (1) in the case of taking drugs that may affect sugar metabolism in the last four weeks (Drugs: psychiatric drugs, oral/injection steroids, diuretics, anti-cancer drugs, epilepsy drugs, tuberculosis drugs, contraceptives, estrogen, anti-HIV drugs, immunosuppressants, beta antagonists, anti-hypertensive drugs, licorice, grapefruit, and carbonoxolone); (2) diabetes, chronic renal failure (creatinine clearance < 60 mL/min), liver disease (AST, ALT > 3 times normal upper limit and cirrhosis above a Child–Pugh score of B grade), pancreatitis (acute/chronic), cancer, heart disease (heart failure, myocardial infarction, and arrhythmia); (3) a pregnant woman; (4) those with colds and upper respiratory tract infections on the day of participation in this study; and (5) a person who has at least two relatives with diabetes within his immediate family (parents, children) and siblings. The criteria for selecting the T2D groups are as follows: (1) those who are satisfied with the diagnosis in accordance with the ADA guidelines [1] and (2) a person diagnosed with T2D or undergoing treatment with diabetes medication. T2D exclusion criteria are as follows: (1) a person who is pregnant or breastfeeding, (2) people who are addicted to alcohol or other drugs, and (3) a person diagnosed with a malignant tumor. We recruited patients with T2D and classified them into T2D without insulin treatment (T2D-w/o-insulin) and with insulin treatment (T2D-with-insulin) groups based on whether they received insulin treatment. We considered the T2D-with-insulin group as a group that may have beta cell deficiency. In general, the duration of diabetes in the T2D-with-insulin group is bound to be significantly longer than that in the T2D-w/o-insulin group. Therefore, we selected patients so that there was no significant difference between the T2D-w/o-insulin and T2D-with-insulin groups in various parameters including age, BMI, weight, fasting blood glucose, HbA1c, and lipid profile except for the duration of diabetes. This research received approval from the Institutional Review Boards at Keimyung University Dongsan Medical Center (2018-11-001, approved date 30 November 2018; 2021-09-030, approved date 30 September 2021). The participants provided an informed consent and were divided into the following three groups: normoglycemia (N = 60), T2D not receiving insulin treatment (T2D-w/o-insulin, N = 71), and T2D receiving insulin treatment (T2D-with-insulin, N = 64).

### 2.2. The Measurement of Biochemical Parameters

After 8 h of fasting, an oral glucose tolerance test (GTT) and blood sampling were performed. Plasma adipsin concentrations were measured using immunoassay (Complement Factor D Magnetic Luminex Performance Assay, R&D Systems, Minneapolis, MN, USA) according to the manufacturer’s protocol. Insulin and C-peptide levels were measured using an ELISA kit (R&D Systems, Minneapolis, MN, USA). The 2-h C-peptidogenic index, homeostasis model assessment of insulin resistance (HOMA-IR), and pancreatic beta cell function (HOMA-Beta) were calculated [19,20].

### 2.3. Animal Experiment

C57BL/6 male mice were obtained from Jung-Ang Experimental Animals (Seoul, Republic of Korea). The mice were fed a normal chow diet (ND, Research Diet, New Brunswick, NJ, USA). To obtain an insulin-deficient T2D mouse model, high-fat-fed mice were injected with low-dose streptozotocin (STZ) and nicotinamide (NA) [21]. In brief, 6-week-old C57BL/6 mice were fed a high-fat diet (HFD; 60% kcal fat; Research Diet) for 4 weeks. Then, the mice were injected once with low-dose STZ (150 mg/kg) and NA (1000 mg/kg) and were then fed a HFD continuously for 2 weeks. Despite the radical differences between human conditions and mouse disease models, we used HFD or HFD-STZ-NA mouse models to mimic T2D or T2D with beta cell insufficiency, respectively. Although the sample size for the mouse experiments may not have sufficient sample power, we used a minimum of eight mice per group to achieve statistical significance. All animal experiments were approved by the Keimyung University Institutional Ethics Committee (Daegu, Korea, KM-2020-17R1). At the end of the study, plasma and white adipose tissue (WAT) samples were collected and stored at −80 °C. Embedded tissue blocks were cut into 6 μm sections and stained with hematoxylin and eosin or insulin immunofluorescence. Plasma adipsin concentration was measured using an ELISA kit (Merck Millipore, Burlington, MA, USA). Homogenates of mouse WAT were subjected to SDS-PAGE and were transferred to Immobilon-P membranes. The membranes were probed with specific primary adipsin antibody (Santa Cruz Biotechnology, Dallas, TX, USA). The membranes were incubated with a secondary antibody (MilliporeSigma, MO, USA) conjugated with horseradish peroxidase. The immunoreactive bands were visualized using enhanced chemiluminescence (Amersham Biosciences, Buckinghamshire, UK).

### 2.4. Exploring Genetic Variants of Adipsin Associated with T2D

We used GWAS meta-analysis datasets of the translational human pancreatic islet genotype tissue-expression resource data portal for exploring genetic variants of adipsin [22]. The datasets included 70K for the T2D GWAS, DIAGRAM 1000G, and DIAGRAM Diamante T2D GWAS. To identify the associated traits of T2D and genetic variants, we also used genetic association datasets of the T2D Knowledge Portal.

### 2.5. Statistical Analysis

SPSS version 27.0 (IBM, Armonk, NY, USA) was used for statistical analyses. Comparisons between the two groups were performed using Student’s two-tailed *t*-test, and comparisons between three groups were performed using one-way analysis of variance (ANOVA) followed by Tukey’s post hoc test. For animal experiments, significance was tested using ANOVA with Bonferroni correction to deal with relatively small numbers of samples. The results are expressed as the mean ± standard error. The partial correlation analysis was used after adjusting for age, sex, duration, or BMI. A value of *p* < 0.05 was considered statistically significant.

## 3. Results

### 3.1. The Association of Adipsin with Clinical Parameters in the Subjects

To determine whether adipsin is a marker that reflects the function of pancreatic beta cells, we measured plasma adipsin concentrations in the subjects (Table 1). The subjects were separated into three groups: (1) normoglycemia, (2) T2D without insulin treatment (T2D-w/o-insulin), and (3) T2D treated with insulin (T2D-with-insulin), which may have beta cell insufficiency. We did not obtain fasting plasma insulin concentrations or HOMA indices in the T2D-with-insulin group because of exogenous insulin treatment. Because we could not perform an oral GTT in the normoglycemia group, we did not obtain 2-h glucose, 2-h C-peptide, or 2-h C-peptiogenic index in the normoglycemia group.

There were significant differences in plasma adipsin concentrations (*p* < 0.001) and fasting C-peptide concentrations (*p* < 0.001) between the three groups. The correlation was analyzed using a partial correlation coefficient adjusted for sex, duration of diabetes, and BMI (Table 2). Plasma adipsin concentration was negatively correlated with HbA1c (*p* = 0.006). There was a significant positive correlation between adipsin and fasting serum C-peptide concentrations (*p* < 0.001). These data suggest that adipsin may be a biomarker reflecting pancreatic beta cell function. Because there were differences in several parameters, including fasting blood glucose, blood pressure, and lipid profiles, between the three groups, it was difficult for us to draw firm conclusions about the correlation analysis.

### 3.2. Association Between Adipsin and Beta Cell Function in Diabetic Groups

**Figure 1:**
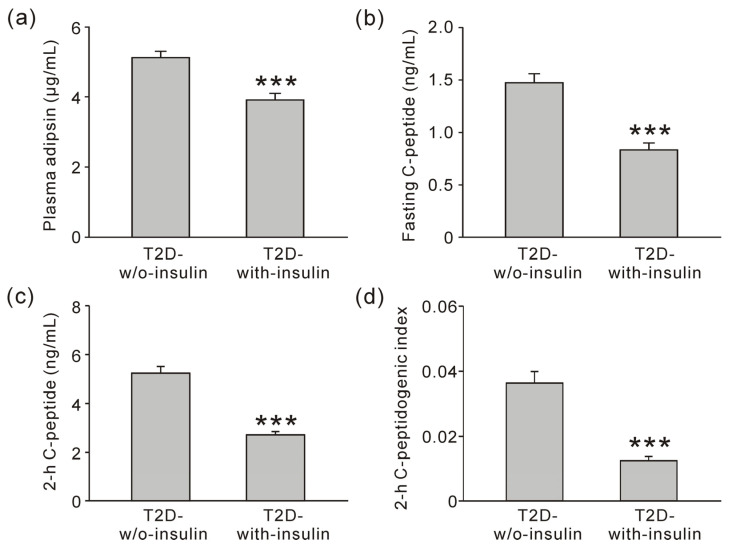
Plasma adipsin concentration varies with pancreatic beta cell function in the diabetic groups. Plasma adipsin concentrations (**a**) and fasting serum C-peptide concentrations (**b**) in the T2D-w/o-insulin (n = 71) and the T2D-with-insulin (n = 64) groups. 2-h C-peptide concentrations (**c**) or 2-h C-pepditogenic index (**d**) during oral glucose tolerance test in the T2D-w/o-insulin and T2D-with-insulin groups. Data represents the mean ± standard error of the mean. *** *p* < 0.001 vs. T2D-w/o-insulin group. T2D, type 2 diabetes; T2D-w/o-insulin, T2D without insulin treatment; T2D-with-insulin, T2D with insulin treatment.

Because many clinical parameters differed significantly between the normal glycemia and other groups, we focused on the two diabetic groups including the T2D-w/o-insulin and the T2D-with-insulin groups to determine whether plasma adipsin concentrations could reflect changes in beta cell function. Between the T2D-w/o-insulin and T2D-with-insulin groups, there were no significant differences in several parameters including BMI, fasting plasma glucose level, blood pressure, and lipid level (Supplementary Table S1). There was also no significant difference in body fat distribution between the two groups (Table 3). However, plasma adipsin concentration (Figure 1a), fasting serum C-peptide concentration (Figure 1b), 2-h C-peptide concentration (Figure 1c), and 2-h C-peptidogenic index (Figure 1d) in the T2D-with-insulin group were significantly decreased compared with the T2D-w/o-insulin group.

Correlations between plasma adipsin concentrations and clinical parameters were analyzed using partial correlation coefficients adjusted for duration of diabetes (Table 4). A statistically negative correlation was identified between adipsin and either 2-h glucose concentration (*p* = 0.026) or HbA1c (*p* = 0.001). Adipsin also exhibited a positive correlation with fasting serum C-peptide (*p* < 0.001), 2-h C-peptide concentration (*p* < 0.001), and 2-h C-peptidogenic index (*p* < 0.001). To confirm the independence of adipsin as a biomarker for beta cell function, we performed a multiple regression analysis of adipsin with various parameters in the T2D-w/o-insulin and the T2D-with-insulin groups. Multiple regression analysis showed that duration of diabetes (*p* = 0.016), fasting C-peptide concentration (*p* = 0.033), 2-h C-peptide concentration (*p* = 0.045), and 2-h C-peptidogenic index (*p* = 0.035) were independently related to plasma adipsin concentration (Table 5). These data suggest that adipsin can be used as a biomarker to evaluate beta cell function in patients with T2D, even in those receiving insulin treatment.

### 3.3. The Assessment of Adipsin Expression and Pancreatic Beta Cell Mass in T2D Mice

**Figure 2:**
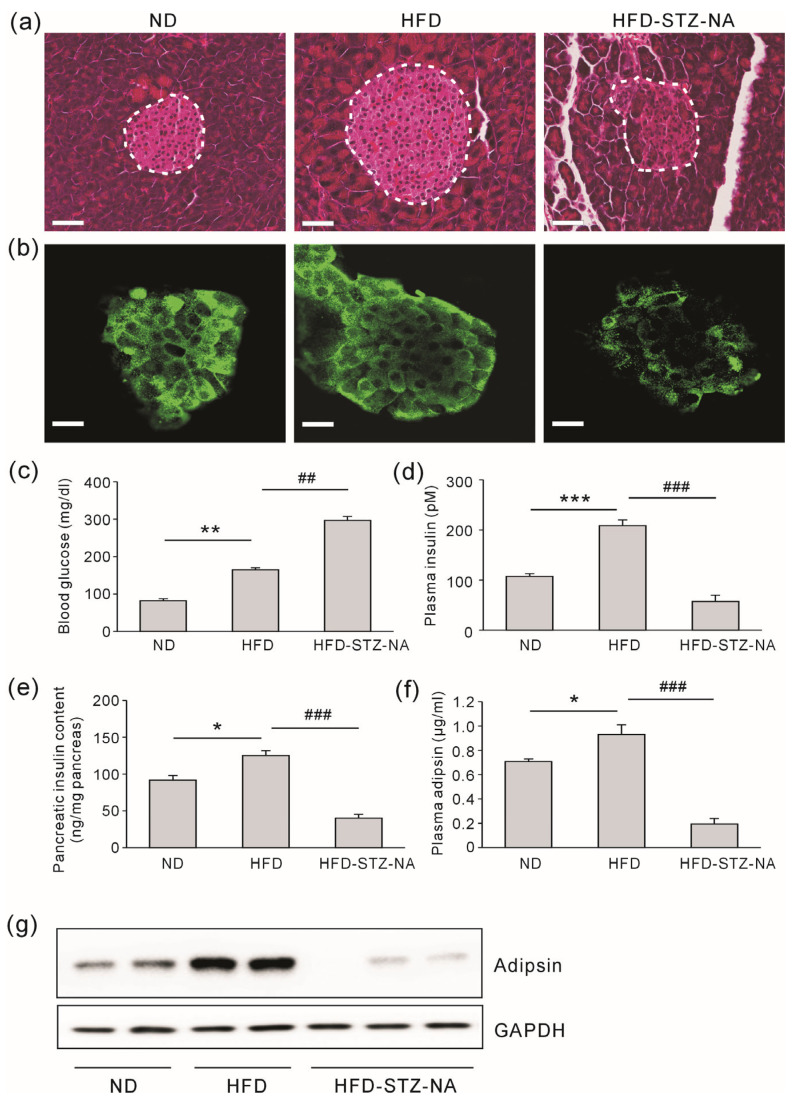
Plasma adipsin concentration varies with pancreatic beta cell function in the diabetic mice. (**a**) Representative images showing the pancreatic islets via hematoxylin and eosin staining (200×). (**b**) Immunofluorescence staining for insulin (green) in mouse pancreatic islets. Scale bars, 50 μm. (**c**) Blood glucose concentration after 10 h of fasting. (**d**) Fasting plasma insulin concentrations. (**e**) Pancreatic insulin contents. (**f**) Plasma adipsin concentrations. Data represent the mean ± standard error of the mean, n = 8. * *p* < 0.05, ** *p* < 0.01, and *** *p* < 0.001 vs. ND group; ## *p* < 0.01 and ### *p* < 0.001 vs. HFD group. (**g**) Protein expression levels of adipsin in WAT of mice were detected via Western blot analysis. ND, normal diet; HFD, high-fat diet; HFD-STZ-NA, high-fat diet with streptozotocin and nicotinamide.

To explore the relationship between adipsin expression and pancreatic beta cell function in animal models comparable to the study group, we utilized ND, HFD, and HFD-STZ-NA mice. Although animal models of T2D are fundamentally different from the human condition, we used the HFD-STZ-NA mouse model to mimic the T2D-with-insulin group [21]. The HFD mice exhibited an increase in pancreatic beta cell mass compared to the ND mice, whereas the insulin-deficient T2D model, HFD-STZ-NA, showed a reduction in beta cell mass compared to the HFD mice (Figure 2a,b). After a 10 h fast, blood glucose levels were significantly higher in the HFD mice compared to the ND mice and further elevated in the HFD-STZ-NA mice compared to HFD (Figure 2c). As expected, plasma insulin concentration, pancreatic insulin content, and plasma adipsin levels were significantly higher in the HFD mice compared to the ND mice and significantly lower in the HFD-STZ-NA mice compared to the HFD mice (Figure 2d–f). Similarly, adipsin expression in WAT followed the same pattern, with a notable increase in the HFD mice compared to the ND mice and a significant decrease in the HFD-STZ-NA mice compared to the HFD mice (Figure 2g). These findings suggest that changes in adipsin expression in WAT correspond to changes in beta cell mass and function in T2D.

### 3.4. The Exploration of T2D-Associated Genetic Variants in Adipsin

To determine the importance of adipsin in the development of T2D, we explored genetic variants of adipsin (single-nucleotide polymorphisms (SNPs)) associated with T2D using GWAS meta-analysis datasets. Three different datasets were searched (Diamante T2D GWAS, Diamante Multi-ancestry T2D GWAS, and 70K for T2D GWAS). We found rs3761010 (sample size = 201,120, *p* < 0.001), rs2965285 (sample size = 332,320, *p* < 0.001), and rs351992 (sample size = 201,750, *p* = 0.002) as T2D-associated genetic variants in adipsin (Table 6). Next, we investigated T2D-related characteristics for rs3761010, rs2965285, and rs351992 using the T2D Knowledge Portal database (Table 7). They were significantly associated with T2D (*p* = 0.001). These data suggest that genetic variants of adipsin are associated with the development of T2D. Therefore, measuring adipsin SNP in pre-diabetic patients can predict the risk of developing T2D.

## 4. Discussion

Since beta cell insufficiency is one of the important factors in the progression of T2D [23,24], the development of reliable markers of pancreatic beta cell function is needed. To date, various indicators have been utilized to evaluate beta cell conditions. However, using these markers comes with certain limitations. The GTT reflects the immediate insulin response of pancreatic beta cells to a glucose challenge; however, it is rather complicated and time-consuming [25]. In contrast, HbA1c represents long-term glycemic status over the preceding 3-month period, partially indicating pancreatic beta cell function without an acute reflection of glycemic levels [26]. New indices such as the HOMA, C-peptide, and C-peptidogenic index have been employed to assess pancreatic function or insulin-secreting capacity [27,28,29,30]. Insulin-based markers such as HOMA-beta are not recommended for patients on exogenous insulin therapy because they cannot measure plasma insulin levels due to exogenous insulin [27]. In addition, complex procedures such as oral glucose tolerance tests are required, which can make it difficult to measure HOMA-beta markers [28,29]. C-peptide, a cleaved product of proinsulin, has been identified as being more reliable for beta cell function. It is slowly degraded and negligibly hepatically eliminated compared to endogenous insulin and is applicable for insulin-dependent cases. However, if large quantities of anti-insulin antibodies are present in the bloodstream, they bind to C-peptide and result in a falsely elevated C-peptide result [30]. Additionally, in diabetes-independent cardiovascular diseases, C-peptide levels tend to increase [31,32]. Due to this false increase in C-peptide, C-peptide may sometimes not accurately reflect beta cell function. Therefore, there is a need to develop simple and reproducible biomarkers that can accurately represent pancreatic beta cell function or status, which can overcome the shortcomings of HOMA-beta and C-peptide in patients with T2D.

We propose that plasma adipsin, which is secreted from adipocytes [10,14], is a potentially novel indicator of beta cell function even in patients with T2D who are undergoing insulin therapy. Therefore, as a biomarker of pancreatic beta cells, adipsin may overcome some of the shortcomings of the HOMA-beta index. Our results showed that plasma adipsin concentration in patients was significantly increased in the T2D-w/o-insulin group compared with the normoglycemia group, and in mice, plasma adipsin concentration and adipsin expression in WAT were significantly increased in the HFD group compared with the ND group. The increased adipsin showed a pattern similar to the increased beta cell mass and insulin secretion due to insulin resistance. However, there are several limitations in concluding that plasma adipsin concentration is related to pancreatic beta cell function because many factors, including body weight, differ between these two groups. A recent study showed that plasma adipsin concentration was associated with obesity [33], and that plasma adipsin concentrations may increase as BMI increases. Interestingly, when we compared the two groups of patients with the same BMI, the T2D-w/o-insulin and the T2D-with-insulin groups, we found that the T2D-with-insulin group had lower plasma adipsin concentrations than the T2D-w/o-insulin group. In addition, we showed that there was no difference in fat distribution, including VAT and SAT, between the T2D-w/o-insulin and the T2D-with-insulin groups. Through correlation analysis and multiple regression analysis, we confirmed that plasma adipsin concentration was related with C-peptide concentration and C-peptidogenic index in the diabetic groups. Therefore, adipsin can be a biomarker of pancreatic beta cells that can compensate for shortcomings such as a false increase in C-peptide. In addition, in a diabetic mouse model, we confirmed that adipsin expression in adipose tissue showed the same pattern of changes in pancreatic beta cell mass and function. However, due to the small number of animal experiments, we could not analyze the correlation between adipsin and pancreatic function in animal experiments.

We have not elucidated the direct role of adipsin in maintaining pancreatic beta cell function and mass. However, recent studies have elucidated the mechanism by which adipsin regulates insulin secretion from pancreatic beta cells through the c3a receptor [17] and the mechanism by which long-term adipsin treatment maintains beta cell mass in diabetic mice through a decrease in intracellular Dusp26 [18]. Additionally, we recently demonstrated that ER stress is an important factor in reducing adipsin expression in adipocytes in a T2D mouse model [34]. These results suggest that changes in adipose tissue can affect pancreatic function and mass through adipsin. These results also suggest that adipsin may be an important target for the treatment of T2D, as well as a biomarker reflecting the function or status of pancreatic beta cells.

In humans, adipsin is encoded by the CFD gene. In the present study, we identified three SNPs in the adipsin region of chromosome 19 associated with T2D: rs3761010, rs2965285, and rs351992. With sample sizes ranging from approximately 200,000 to 330,000, these were statistically significantly associated with T2D. Specifically, after adjusting for BMI, rs3761010 was not associated with insulin sensitivity but was significantly associated with T2D. Patients with these adipsin variants may be at higher risk of eventually developing T2D. Genetic testing for these adipsin SNPs may provide early information on the onset and progression of T2D and may help predict future changes in a patient’s beta cell function.

The present study has some limitations. First, we could not investigate the correlation between plasma adipsin level and 30 min or 60 min C-peptidogenic index because of sampling restrictions. Second, the imbalance of gut microbiome has been reported as a major cause of T2D [35]. The dysbiosis of gut microbiota can affect adipocytes through systemic inflammation, metabolic changes, insulin resistance, etc. However, the effect of dysbiosis on adipsin expression of adipocytes is not known yet, and further studies are needed. Third, adipsin clearance occurs mainly through the renal system; thus, its circulating level can be affected by urinary diseases, especially renal failure [36,37]. In our study, all participants had normal kidney function. Fourth, we did not consider all the various factors such as the effectiveness of disease management, diet, and physical activity in the diabetic group. However, all subjects were selected as non-smokers, and patients using the same class of drugs were recruited as much as possible. Finally, our study did not monitor adipsin levels during the progression of diabetes or pancreatic beta cell dysfunction. Therefore, we clinically divided patients with T2D into the T2D-w/o-insulin and the T2D-with-insulin groups to assess their insulin secretory capacity. Therefore, additional long-term prospective studies or further analyses of large numbers of patients are needed. In particular, longitudinal studies focusing on tracking adipsin levels as diabetes progresses would be valuable. For adipsin to become an accurate and effective biomarker for predicting the function of beta cells, its relationship with various cytokines, such as inflammatory cytokines or other adipokines, may need to be considered, and further analysis is needed.

## 5. Conclusions

We investigated the association between plasma adipsin levels and pancreatic beta-cell function in diabetic patients and mice. We showed that plasma adipsin levels can reflect the function of pancreatic beta cells in diabetic patients receiving insulin treatment. Through clinical and genetic analyses as well as animal experiments, we highlighted the importance of adipsin as a biomarker capable of assessing beta cell function in patients with T2D.

## Figures and Tables

**Table 1:** Baseline characteristics of the subjects.

	Normoglycemia (n = 60)	T2D-w/o-Insulin (n = 71)	T2D-with- Insulin (n = 64)	*p*-Value
Sex (Male/Female)	19/41	46/35	38/26	0.003
Age (year)	57.05 ± 7.59	57.48 ± 10.93	57.86 ± 12.04	0.662
Duration of diabetes (year)	0	7.02 ± 6.30	15.41 ± 9.18	<0.001
BMI	22.13 ± 1.53	25.04 ± 3.88	25.64 ± 4.02	<0.001
Body weight (kg)	57.02 ± 7.48	66.77 ± 13.93	68.05 ± 13.09	<0.001
Fasting glucose (mg/dL)	88.95 ± 5.99	122.31 ± 27.01	125.28 ± 42.74	<0.001
HbA1c (%)	5.41 ± 0.17	7.07 ± 1.10	8.88 ± 2.21	<0.001
Fasting insulin (μIU/mL)	4.01 ± 2.27	8.69 ± 5.19	-	-
Fasting C-peptide (ng/mL)	0.91 ± 0.32	1.47 ± 0.73	0.83 ± 0.54	<0.001
HOMA-IR	0.84 ± 0.49	2.77 ± 2.52	-	-
HOMA-beta (%)	35.15 ± 20.30	61.45 ± 52.24	-	-
2-h ^1^ glucose (mg/dL)	-	262.40 ± 72.05	304.92 ± 72.52	-
2-h C-peptide (ng/mL)	-	5.24 ± 2.32	2.72 ± 1.07	-
2-h C-peptidogenic index	-	0.04 ± 0.03	0.01 ± 0.01	-
Plasma adipsin (μg/mL)	4.53 ± 1.15	5.11 ± 1.53	3.91 ± 1.51	<0.001
Systolic blood pressure (mmHg)	119.63 ± 11.55	125.19 ± 17.72	125.42 ± 14.34	0.052
Diastolic blood pressure (mmHg)	74.77 ± 8.22	69.43 ± 11.60	71.09 ± 10.46	0.053
AST (U/L)	21.05 ± 4.95	25.24 ± 18.53	22.80 ± 8.34	0.062
ALT (U/L)	18.42 ± 7.91	23.60 ± 22.08	22.91 ± 14.94	0.058
Creatinine (mg/mL)	0.72 ± 0.14	0.85 ± 0.30	0.87 ± 0.46	0.021
eGFR (mL/min/1.73 m^2^)	101.20 ± 17.04	90.17 ± 24.85	95.57 ± 34.84	0.062
Triglyceride (mg/dL)	108.21 ± 50.66	144.84 ± 111.00	148.19 ± 106.62	0.036
HDL (mg/dL)	55.21 ± 11.98	49.42 ± 13.54	44.38 ± 9.93	0.062
LDL (mg/dL)	112.33 ± 32.20	87.75 ± 34.51	78.39 ± 24.97	<0.001

Values are expressed as number or mean ± standard deviation. BMI, body mass index; HbA1c, Hemoglobin A1c; HOMA-beta, homeostasis model assessment of beta cell function; HOMA-IR, homeostatic model assessment of insulin resistance; T2D, type 2 diabetes; T2D-w/o-insulin, T2D without insulin treatment; T2D-with-insulin, T2D with insulin treatment. ^1^ Serum glucose and C-peptide concentrations were measured after 2-h using an oral glucose tolerance test.

**Table 2:** Correlation ^1^ between adipsin and clinical parameters of the subjects.

	Partial Correlation R	Partial Correlation *p*
HbA1c (%)	−0.196	0.006
Fasting C-peptide (ng/mL)	0.272	<0.001

^1^ Partial correlation coefficient adjusted for sex, duration, and BMI. HbA1c, Hemoglobin A1c.

**Table 3:** Body composition of the diabetic groups.

	T2D-w/o-Insulin	T2D-with-Insulin	*p*-Value
BMI	24.98 ± 0.45	25.65 ± 0.51	0.331
Waist (cm)	86.89 ± 1.00	88.32 ± 1.16	0.116
VAT area (cm^2^)	146.33 ± 6.70	151.89 ± 7.28	0.124
SAT area (cm^2^)	19.74 ± 0.32	20.30 ± 0.35	0.247
Visceral to subcutaneous fat ratio (VSR)	6.94 ± 0.31	7.59 ± 0.39	0.132
Total body fat mass (kg)	23.40 ± 1.20	24.76 ± 1.31	0.443
Total body fat percentage (%)	33.54 ± 0.69	33.78 ± 0.90	0.836
Total body lean mass (kg)	41.15 ± 1.10	43.19 ± 1.05	0.192

BMI, body mass index; SAT, subcutaneous adipose tissue; T2D, type 2 diabetes; T2D-w/o-insulin, T2D without insulin treatment; T2D-with-insulin, T2D with insulin treatment; VAT, visceral adipose tissue.

**Table 4:** Correlation ^1^ between adipsin and clinical parameters in the diabetic groups.

	Partial Correlation R	Partial Correlation *p*
2-h ^2^ glucose (mg/dL)	−0.187	0.026
HbA1C (%)	−0.277	0.001
Fasting C-peptide (ng/mL)	−0.321	<0.001
2-h C-peptide (ng/mL)	−0.384	<0.001
2-h C-peptidogenic index	−0.363	<0.001

HbA1c, Hemoglobin A1c. ^1^ Partial correlation coefficient adjusted for duration. ^2^ Serum glucose and C-peptide concentrations were measured after 2-h using an oral glucose tolerance test.

**Table 5:** Adipsin with clinical parameters of the multiple regression analysis in the diabetic groups.

	Unstandardized Coefficients		Standardized Coefficients	t	*p*
	B	SE	B		
(constant)	3.813	0.945		4.034	0.000
Duration of diabetes (year)	0.045	0.019	0.230	2.443	0.016
Fasting glucose (mg/dL)	0.001	0.006	0.019	0.147	0.883
2-h ^1^ glucose (mg/dL)	0.002	0.003	0.104	0.781	0.436
HbA1C (%)	−0.110	0.080	−0.125	−1.386	0.168
Fasting C-peptide (ng/mL)	0.580	0.269	0.259	2.151	0.033
2-h C-peptide (ng/mL)	0.020	0.115	0.027	0.170	0.045
2-h C-peptidogenic index	18.478	8.671	0.320	2.131	0.035

B, regression coefficient; HbA1c, Hemoglobin A1c; SE, standard error. ^1^ Serum glucose and C-peptide concentrations were measured after 2-h using an oral glucose tolerance test.

**Table 6:** The top adipsin variants at ±100 Kb and their associations with T2D.

SNP ID	Chromosome	Location	Reference Allele	Alternate Allele	Sample Size	*p*-Value
Diamante T2D GWAS rs3761010	19	19:849,935	A	C	201,120	<0.001
Diamante Multi-ancestry T2D GWAS rs2965285	19	19:896,506	A	G	332,320	<0.001
70K for T2D GWAS rs351992	19	19:813,979	C	T	201,750	0.002

GWAS, Genome-wide association study; T2D, type 2 diabetes.

**Table 7:** Association of the top adipsin variants across all datasets and traits included in the T2D Knowledge Portal.

Phenotype	*p*-Value	Odds Ratio	Sample Size
rs3761010			
T2D adjust BMI	0.001	▼0.9719	249,271
T2D	0.016	▼0.9925	1,243,220
rs2965285			
T2D adjust BMI	<0.001	▲1.0454	253,256
T2D	<0.001	▲1.0223	893,509
rs351992			
T2D adjust BMI	0.024	▼0.9768	249,541
T2D	0.044	▼0.9907	1242,410

T2D, type 2 diabetes. ▲ depicts an increased value, ▼ depicts a decreased value.

## Data Availability

The original contributions presented in the study are included in the article, further inquiries can be directed to the corresponding author.

## Supplementary Materials

The following supporting information can be downloaded at the following website: https://www.mdpi.com/article/10.3390/jcm13237351/s1, Table S1: Baseline characteristics in the diabetic groups.
