# Supplementary material for: Identification of Adipsin as a Biomarker of Beta Cell Function in Patients with Type 2 Diabetes
Source: J Clin Med. 2024 Dec 2;13(23):7351. doi: 10.3390/jcm13237351 (PMC11642166; doi:10.3390/jcm13237351)
Supplement: Supplementary file 1 [file jcm-13-07351-s001.zip › jcm-3310633-supplementary.pdf]

Supplementary Table S1. Baseline characteristics in the diabetic groups

	T2D (n=71)	T2D with insulin (n=64)	P-value
Sex (Male/Female)	46/35	38/26	0.754
Age (year)	57.48 ± 10.93	57.86 ± 12.04	0.844
Duration of diabetes (year)	7.02 ± 6.30	15.41 ± 9.18	<0.001
BMI	25.04 ± 3.88	25.64 ± 4.02	0.526
Body weight (kg)	66.77 ± 13.93	68.05 ± 13.09	0.481
Fasting glucose (mg/dL)	122.31 ± 27.01	125.28 ± 42.74	0.629
HbA1c (%)	7.07 ± 1.10	8.88 ± 2.21	0.059
Fasting insulin (μIU/mL)	8.69 ± 5.19	-	-
Fasting C-peptide (ng/mL)	1.47 ± 0.73	0.83 ± 0.54	<0.001
HOMA-IR	2.77 ± 2.52	-	-
HOMA-Beta	61.45 ± 52.24	-	-
2 h[1] glucose (mg/dL)	262.40 ± 72.05	304.92 ± 72.52	0.001
2 h C-peptide (ng/mL)	5.24 ± 2.32	2.72 ± 1.07	<0.001
2 h C-peptidogenic index	0.0366 ± 0.03	0.0127 ± 0.01	<0.001
Plasma adipsin (μg/mL)	5.11 ± 1.53	3.91 ± 1.51	<0.001
Systolic blood pressure (mmHg)	125.19 ± 17.72	125.42 ± 14.34	0.726
Diastolic blood pressure (mmHg)	69.43 ± 11.60	71.09 ± 10.46	0.538
AST (U/L)	25.24 ± 18.53	22.80 ± 8.34	0.465
ALT (U/L)	23.60 ± 22.08	22.91 ± 14.94	0.792
Creatinine (mg/mL)	0.85 ± 0.30	0.87 ± 0.46	0.584
eGFR (mL/min/1.73m^2)	90.17 ± 24.85	95.57 ± 34.84	0.073
Triglyceride (mg/dL)	144.84 ± 111.00	148.19 ± 106.62	0.375
HDL (mg/dL)	49.42 ± 13.54	44.38 ± 9.93	0.195
LDL (mg/dL)	87.75 ± 34.51	78.39 ± 24.97	0.083

Values are expressed as number or mean ± standard deviation. ALT, alanine aminotransferase; AST, aspartate aminotransferase; BMI, body mass index; eGFR, estimated glomerular filtration rate; HDL, high-density lipoprotein; HOMA-Beta, homeostasis model assessment of beta cell function; HOMA-IR, homeostatic model assessment of insulin resistance; LDL, low-density lipoprotein; T2D, type 2 diabetes without insulin treatment; T2D with insulin, type 2 diabetes with insulin treatment.

[1] Serum glucose and C-peptide concentrations were measured after 2 hours using an oral glucose tolerance test
